# Noble Metal Nanoparticle-Loaded Porphyrin Hexagonal Submicrowires Composites (M-HW): Photocatalytic Synthesis and Enhanced Photocatalytic Activity

**DOI:** 10.3390/nano13040660

**Published:** 2023-02-08

**Authors:** Shuanghong Liu, Guan Huang, Jiefei Wang, Jianshuai Bao, Mengyue Wang, Yaqun Wei, Yong Zhong, Feng Bai

**Affiliations:** 1 Key Laboratory for Special Functional Materials of Ministry of Education, National & Local Joint Engineering Research Center for High-Efficiency Display and Lighting Technology, School of Materials Science and Engineering, Collaborative Innovation Center of Nano Functional Materials and Applications, Henan University, Kaifeng 475004, China; 2 International Joint Centre for Biomedical Innovation, School of Life Sciences, Henan University, Kaifeng 475004, China

**Keywords:** porphyrin, self-assemblies, surface plasmon resonance, photocatalytic

## Abstract

Surface plasmon resonance (SPR) photocatalysts have attracted considerable attention because of their strong absorption capacity of visible light and enhanced photogenic carrier separation efficiency. However, the separate production of metal nanoparticles (NPs) and semiconductors limits the photogenic charge transfer. As one of the most promising organic photocatalysts, porphyrin self-assemblies with a long-range ordered structure-enhance electron transfer. In this study, plasmonic noble metal-based porphyrin hexagonal submicrowires composites (M-HW) loaded with platinum (Pt), silver (Ag), gold (Au), and palladium (Pd) NPs were synthesized through a simple in situ photocatalytic method. Homogeneous and uniformly distributed metal particles on the M-HW composites enhanced the catalytic or chemical properties of the organic functional nanostructures. Under the same loading of metal NPs, the methyl orange photocatalytic degradation efficiency of Ag-HW [k_Ag-HW_ (0.043 min^−1^)] composite was three times higher than that of HW, followed by Pt-HW [k_Pt-HW_ (0.0417 min^−1^)], Au-HW [k_Au-HW_ (0.0312 min^−1^)], and Pd-HW [k_Pd-HW_ (0.0198 min^−1^)]. However, the rhodamine B (RhB) and eosin B photocatalytic degradations of Pt-HW were 4 times and 2.6 times those of HW, respectively. Finally, the SPR-induced electron injection, trapping, and recombination processes of the M-HW system were investigated. These results showed that M-HW plasmonic photocatalysts exhibited excellent photocatalytic performances, making them promising materials for photodegrading organic pollutants.

## 1. Introduction

Environmental pollution has become a major concern because of rapid industrial development, which greatly threatens human health and social stability [1,2,3]. As a result, photocatalysis, as an ideal environmental purification and green energy production technology using semiconductor materials, has attracted considerable scholarly attention. In addition, it uses solar energy for the photodegradation of organic pollutants [4], organic synthesis (e.g., selective oxidation and reduction), and water splitting for hydrogen generation. Therefore, the synthesis of efficient photocatalysts is crucial. The two key factors that determine the catalytic efficiency are the absorption spectrum and separation efficiency of the photogenerated charge of the materials [5]. Noble metal (such as Au, Pt, Ag, and Pd) nanoparticles (NPs) can strongly absorb visible light owing to their surface plasmon resonance (SPR) features [6,7,8,9]. They can also enhance the separation of a photogenerated charge over the Schottky barrier, thereby greatly improving the photocatalytic and photovoltaic performance. In general, NPs have a wide application in colorimetric sensors, photovoltaic devices, photochromic devices, and photocatalysis [10,11,12].

Recently, studies have focused on the control of shapes and sizes of metal NPs for the synthesis of semiconductor-based plasmonic photocatalysts [13,14,15]. Several chemical or photoreduction processes have been developed for the synthesis of SPR composite structures, despite the limited control of the size and spatial distribution of the metal NPs [16,17]. The incorporations of plasmonic Au or Ag nanoparticles (NPs) into wide-bandgap semiconductors, such as titanium dioxide (TiO_2_) and cerium dioxide (CeO_2_), have enhanced the photocatalytic activities in the visible light region [18,19,20,21,22]. In some cases, the separate production of metal NPs and semiconductor NPs can limit the charge transfer [23,24]. Therefore, it is of great technical interest to develop a simple and efficient method for the synthesizing of SPR nanocomposites with precise dimensional control and a wide absorption spectrum, which may have important implications for solar energy harvesting. The reduced particle size greatly enhances the surface-to-volume ratio of the catalysts, thus improving the catalytic performance of noble metals [25,26].

Nevertheless, finding a catalyst with a broad absorption spectrum is crucial [27,28,29]. Porphyrins have a similar structure as chlorophyll (found in plants, which is essential for the photosynthesis of the plant) with a good light sensitivity due to their large π-conjugated system [30,31,32]. Self-assembled porphyrin nanostructures formed through a noncovalent bond interaction can exhibit inherit electronic and optical properties with a similar broadened optical absorption as those of the original porphyrin monomers [33,34,35]. Porphyrin-order nanostructures have attracted widespread attention as a promising photovoltaic technology aiming to emulate the natural light-harvesting processes and energy storage [35,36,37]. After the light absorption process of the self-assembled porphyrin, it becomes excited through the visible light. Then, the well-defined self-assembled porphyrin networks can be combined with molecular oxygen to produce reactive oxygen species (ROS) for photodegrading organic pollutants in the environment [38]. As the energy transfer among porphyrin molecules and the delocalization of excited state electrons strongly depends on the molecular packing mode, we reported the morphology-dependent photocatalytic performance of the porphyrin nanocrystals [34]. Another study has reported a facile photocatalytic process to synthesize and tune the well-defined hollow Pt nanostructures through hierarchically structured templates, where the size of the Pt nanoparticles on the surface of porphyrin nanocrystals ranges from 3.5 to 4.2 nm [39]. This study has provided an important approach for synthesizing organic-based SPR photocatalysts with the controllable size and spatial distribution of the metal NPs.

**Figure 1:**
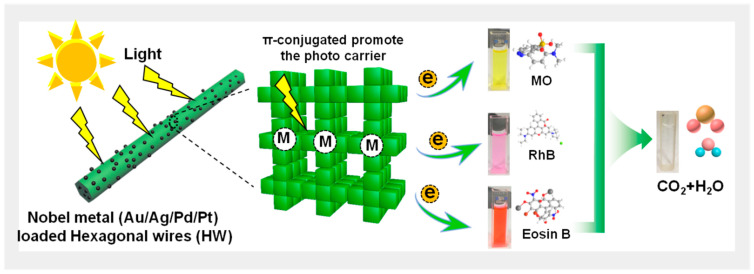
The schematic image of the photodegradation of different organic pollutants using one-dimensional M-HW nanocomposites.

In this work, we proposed and developed a simple in situ photocatalytic synthesis method to load the metal NPs (Pt, Ag, Au, and Pd) on porphyrin hexagonal submicrowires (HW) to form SPR organic composites (M-HW). Then, the M-HW was used to photodegrade methyl orange (MO), rhodamine B (RhB), and eosin B (Figure 1). Owing to the Schottky junction, the M-HW nanocomposites exhibited the enhanced photocatalytic degradation of organic dyes compared with the HW. The SPR-induced electron injection, trapping, and recombination processes of the M-HW system were investigated. 

## 2. Materials and Methods

### 2.1. Reagents and Materials

Cetyltrimethylammonium bromide (CTAB) was obtained from Sigma-Aldrich, and 5, 10, 15, 20-tetrakis (4-pyridyl) zinc porphyrin (ZnTPyP) was purchased from Frontier Scientific. Sodium hydroxide standard solution (NaOH, 1 N) was obtained from Acros, and hydrochloric acid (HCl) was obtained from Kaifeng Dongda Chemical Co., Ltd. Methyl orange (MO) and ascorbic acid (AA) were purchased from Aladdin. Rhodamine B (RhB) and eosin B were obtained from J&K. *N,N*-dimethylformamide (DMF) was obtained from Tianjin Deen Chemical Reagent Co., Ltd. Tetra-n-butyl hexafluorophosphate (TBAPF_6_) was obtained from Alfa Aesar. All the solutions were prepared with ultrapure water from a Barnstead Nanopure water system (resistivity of 18.2 MΩ·cm).

### 2.2. Synthesis of M-HW Nanocomposites

The synthesis of the ZnTPyP hexagonal submicrowires (HW): HW were prepared via an acid-based neutralization surfactant encapsulation self-assembly method [39,40]. Typically, 0.5 mL of ZnTPyP (0.01 M) solution was quickly injected into 9.5 mL of an aqueous solution containing cetyltrimethylammonium bromide (CTAB) (0.01 M) and NaOH (0.02 M) and was rapidly stirred at room temperature (25 °C) for 48 h. The ZnTPyP hexagonal HW was centrifuged at 9500 rpm and washed three times with water to remove the surfactant. Then, the HW was redispersed in water with a mass concentration of 6.67 mg/mL for preparing metal-organic composites (M-HW).

The synthesis of Pt-HW composite: K_2_PtCl_4_ (10 mM) solution and an equal volume of AA solution (0.1 M) were added to a 20 mL glass vial containing 10 mL of dispersed HW (0.1 mg/mL). The mixture was sonicated to ensure it was homogenized and it was then irradiated with a photocatalytic system at room temperature. All the photocatalytic reactions were irradiated using a 300 W xenon lamp equipped with a UVCUT400 filter (λ ≥ 400 nm), in which the incident light passed through a 10 cm water filter. The center of the glass vial was about 20 cm away from the light source, and the light intensity was approximately 100 mW/cm^2^. After the mixture was irradiated for 30 min, the solution changed from purple to black under visible light, indicating that the Pt metal was reduced. After the solution was centrifuged at 9500 rpm for 10 min, monodisperse Pt-HW hybrid material was collected. By adjusting the volume of K_2_PtCl_4_ solution with 10 μL (2.0% Pt-HW), 30 μL (5.6% Pt-HW), 50 μL (9.1% Pt-HW), 100 μL (16.7% Pt-HW), 200 μL (28.6% Pt-HW), 400 μL (45% Pt-HW), and the corresponding volume of AA solution, Pt-HW composites with different platinum loading were obtained.

The preparation of the Ag-HW composite: Ag-HW composites with different silver loadings were obtained by replacing K_2_PtCl_4_ solution with 20 mM silver(I) thiosulfate solution and adjusting the volume of Na_3_Ag(S_2_O_3_)_2_ solution with 30 μL (5.6% Ag-HW), 50 μL (9.1% Ag-HW), 70 μL (12.3% Ag-HW), 150 μL (23% Ag-HW), and 400 μL (45% Ag-HW). The corresponding volume of the AA solution was the same as the preparation method of the Pt-HW composites. Na_3_Ag(S_2_O_3_)_2_ solution was prepared according to a published procedure [41,42].

The preparation of the Pd-HW composite: Pd-HW composites with different palladium loading were prepared by replacing K_2_PtCl_4_ solution with 10 mM of Na_2_PdCl_4_ solution and adjusting the volume of Na_2_PdCl_4_ solution with 60 μL (5.6% Pd-HW), 100 μL (9.1% Pd-HW), 200 μL (16.7% Pd-HW), and 400 μL (28.6% Pd-HW) and the corresponding volume of AA solution.

The preparation of the Au-HW composite: Au-HW composites with different Au loadings were obtained by replacing the K_2_PtCl_4_ solution with 10 mM of Au(I) thiourea solution and adjusting the volume of Au(I) thiourea solution with 30 μL (5.6% Au-HW), 50 μL (9.1% Au-HW), 100 μL (16.7% Au-HW), and 200 μL (28.6% Ag-HW), and the corresponding volume of AA solution was the same to the preparation method of Pt-HW composites. Au(I) thiourea solution was freshly prepared according to a published procedure [43].

### 2.3. Photocatalytic Activity

The photocatalytic activity of M-HW was evaluated through the photocatalytic degradation of the organic pollutants. Precisely, 1 mg of M-HW composites (photocatalyst) was added to 40 mL of MO (20 mg/L, target dye). The mixture was stirred continuously in the dark for 30 min to obtain a homogenized mixture and achieve adsorption–desorption equilibrium. Then, the mixture was transferred to the photocatalytic reaction system and irradiated under light at a specific time, and aliquots of 4 mL of the solution were collected in 7 mL Eppendorf (Ep) tubes every 20 min, and the supernatant was removed twice using high-speed centrifugation at 9500 rpm for 10 min. The absorbance value of MO at the maximum absorption wavelength (464 nm) was measured using a visible spectrophotometer (Shanghai Prism Technology 722SP). Different target dyes, such as rhodamine B (1 × 10^−5^ M, λ_max_ = 554 nm) and eosin B (5 × 10^−5^ M, λ_max_ = 518 nm), were changed in the same condition to record the maximum absorption value. The photocatalytic activities of M-HW on different dyes were studied using the photocatalytic degradation curve.

The photocatalytic degradation curve of organic dyes can be fitted using the first-order kinetic Equation (1) as follows:

(1) $$\mathrm{In}\frac{C_{0}}{C}=\mathrm{kt}$$

where C_0_ (mg/mL) and C (mg/mL) represent the initial organic dye concentration and the dye concentration after irradiation, respectively; k represents the apparent rate constants, and t represents the reaction time (min) [44]. Thus, k was used to evaluate the photocatalytic activity in this work.

### 2.4. Electrochemical Measurements

The electrochemical properties of the 0.1 mM ZnTPyP molecule, rhodamine B, MO, and eosin B dichloromethane solution were measured with cyclic voltammetry (CV) using the conventional three-electrode system, in which a 3.0 mm diameter glass carbon disk (0.071 cm^2^, Shanghai Chenhua) was used as the working electrode, a platinum wire was used as the counter electrode, and Ag/AgCl (silver/silver chloride) was used as the reference electrode (3.0 M KCl solution. The electrode potential was +0.22 V vs. the NHE potential, and 0.1 M of tetra-n-butylammonium hexafluorophosphate (TBAPF_6_) was used as the electrolyte. The oxidation-reduction potential was measured through an electrochemical workstation. Based on the optical and electrochemical data, the energy levels of the HOMOs and LUMOs of the organic dyes can be estimated using Equations (2)–(4).

(2) $$E_{g}=\frac{\mathrm{hc}}{\lambda_{\mathrm{abs}}}=\frac{1240}{\lambda_{\mathrm{abs}}}$$

(3) $$E_{\mathrm{HOMO}}=-E_{\mathrm{ox}\;\left(\mathrm{vs}.\;\mathrm{Ag}/\mathrm{AgCl}\right)}-E_{\mathrm{NHE}\;\left(\mathrm{vs}.\;\mathrm{Ag}/\mathrm{AgCl}\right)}-4.5$$

(4) $$E_{\mathrm{LUMO}}=E_{\mathrm{HOMO}}-E_{g}$$

where E_g_ is the energy gap and λ_abs_ is the maximum UV–Vis absorption wavelengths value in the dichloromethane solution. E_HOMO_ and E_LUMO_ are the energy levels of the HOMOs and LUMOs, respectively. The standard hydrogen electrode (NHE) potential was −4.5 eV relative to the vacuum level. The CV curve test ranged from −1.6 V to 1.6 V, and the scan rate was 200 mV/s. All electrochemical tests were performed at 25 °C under N_2_ saturation.

### 2.5. Materials Characterization

High-resolution transmission electron microscope (HRTEM) images were recorded on a JEOL JEM-2100 operating at an acceleration voltage of 200 kV. Field emission scanning electron microscopy (FESEM) images were recorded using a Nova Nano SEM 450 microscope. The UV/Vis spectra measurements of the M-HW dispersion were performed using an Agilent Technologies Cary 60. A 300 W xenon arc lamp (PLS-SXE300/300UV, Beijing Perfectlight Technology Co., Ltd., Beijing, China) installed in a laboratory lamp housing system provided with light power. The CV curves were tested using an [H] electrochemical workstation (Shanghai Chenhua Instrument Co., Ltd., Shanghai, China).

## 3. Results

### 3.1. Morphology and Size Distribution of M-HW Nanocomposites

**Figure 2:**
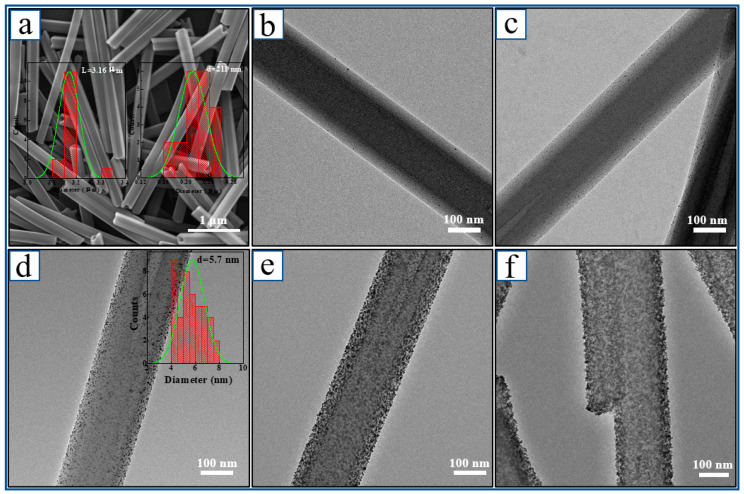
TEM image of one-dimensional M-HW composite: (**a**) porphyrin hexagonal wires (HW) and insert of (**a**) is the size distribution of HW; (**b**) 2.0% Pt-HW; (**c**) 5.6% Pt-HW; (**d**) 9.1% Pt-HW and insert of (**d**) is the size distribution of Pt nanoparticles; (**e**) 23% Pt-HW; (**f**) 45% Pt-HW.

The self-assembled porphyrin networks exhibited excellent photocatalytic activity during the photodegradation of the organic pollutants, photocatalytic water splitting for hydrogen production, and the photocatalytic reduction of metal ions [45,46,47,48]. Thus, owing to the excellent photocatalytic recyclability of ZnTPyP porphyrin self-assembled HWs [49], they were used as an important template to construct organic-based SPR photocatalysts with a controllable size and spatial distribution of the metal NPs. In this study, noble metal (including Au, Ag, Pt, and Pd) nanoparticles were loaded on the surface of HW through a simple in situ photocatalytic reduction method. First, the zinc porphyrin HWs were prepared using the acid-based neutralization surfactant encapsulation the self-assembly method following the procedure of our previous report [34]. The scanning electron microscopy (SEM) image of the HWs showed that the nanostructures were well-defined one-dimensional (1D) submicrowires (Figure 2a) with a hexagonal external profile and a narrow size distribution in both the length and diameter. The average length and the diameter of the submicrowires were 3.16 μm and 211 nm, respectively. Based on the surfactant-assisted self-assembly synthesis process of HW, a thin layer of CTAB surfactants was deposited on the surfaces of HW, resulting in the good dispersibility of the nanomaterials and the adsorption of the metal salt ions. Then, the monodisperse HWs were used for the photocatalytic reduction of K_2_PtCl_4_ under irradiation of visible light in the presence of AA as an electron donor. Through the photocatalytic reaction, K_2_PtCl_4_ was initially in situ reduced to form highly uniform Pt nanoparticles on the surfaces of HW. By changing the volume of the K_2_PtCl_4_ solution and the corresponding volume of the AA solution, Pt-HW composites with different platinum loading were obtained. A transmission electron microscope (TEM) was used to examine the distribution of Pt nanoparticles and the change in their sizes on the surface of submicrowires with Pt loading progression under standard conditions (Figure 2b–f). The results showed that the distribution amount of Pt nanoparticles increased as the amount of feed added increased. In addition, the Pt nanoparticles with precise dimensional control were uniformly supported on the surface of the HWs, and the size was ~5.7 nm by size statistics, as shown in the insert of Figure 2d. When the loading of Pt was below 23 wt%, relative to the amount of HW (Figure 2e), then the monodispersed Pt-HW nanocomposites were obtained by controlling the feed added to the K_2_PtCl_4_ solution. A shell of Pt nanoparticle networks gradually formed on the surface of the photoactive porphyrin nanostructures when the Pt precursors were continuously added to the system. When the loading of Pt was greater than 50 wt%, a shell of Pt nanoparticle networks was formed, which is consistent with the result in the literature [39]. The shell was formed because the cationic CTAB adsorbed on the HW surface played a key role in adsorbing the negatively charged metal salt precursor, in situ photoreducted to the zero-valent metal, which then contributed to the uniform distribution of the metal particles. Such uniformly distributed metal particles enhanced the plasmon resonance and interaction between the electrons, thus contributing to the catalytic or chemical properties of the organic functional nanostructures.

**Figure 3:**
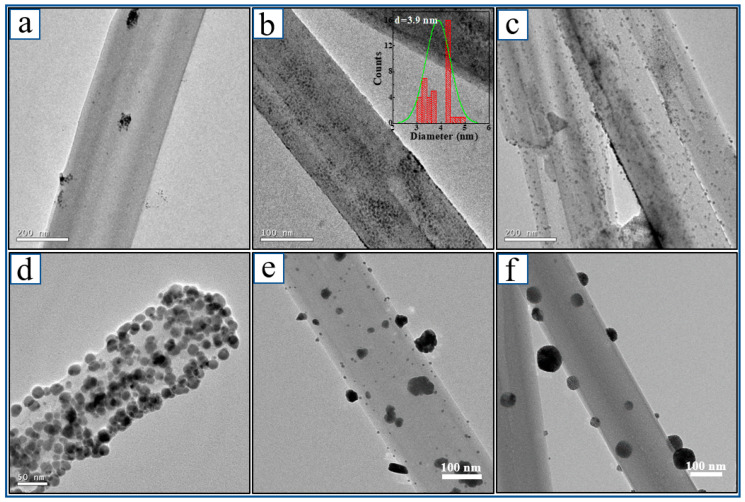
TEM image of one-dimensional M-HW composite: (**a**) 5.6% Ag-HW; (**b**) 12.3% Ag-HW; (**c**) 23% Ag-HW; (**d**) 45% Ag-HW; (**e**) 16.7% Au-HW; (**f**) 9.1% Pd-HW.

In the same way, other noble metal nanoparticles (Ag, Au, and Pd) were successfully deposited on the surface of HW (Figure 3). Ag-HW composites were synthesized by replacing the K_2_PtCl_4_ solution with silver (I) thiosulfate solution (Figure 3a–d). The Ag nanoparticles were in situ reduced and supported on the surface of HW, and the sizes of the Ag nanoparticles were ~3.9 nm (Figure 3b) by size statistics. The loading amounts of different metals were adjusted by regulating the feeding amount of the metal salt precursor. However, Au and Pd NPs did not have such a feature because the strong Ostwald ripening effect facilitated the reduced metal particles to agglomerate during the metal salt precursor reduction process and exposed the HW surfaces [50], which might be related to the growth rate of the crystal faces of various metals and the surface energy. The Au-HW composite materials were shown in Figure 3e. The Au nanoparticles were uniformly supported on the surface of HW, and the Au particle size was ~15 nm by the size statistics. The Pd particle size of the Pd-HW composite was ~20 nm by the size statistics (Figure 3f).

### 3.2. UV–Vis Absorption of M-HW Nanocomposites

**Figure 4:**
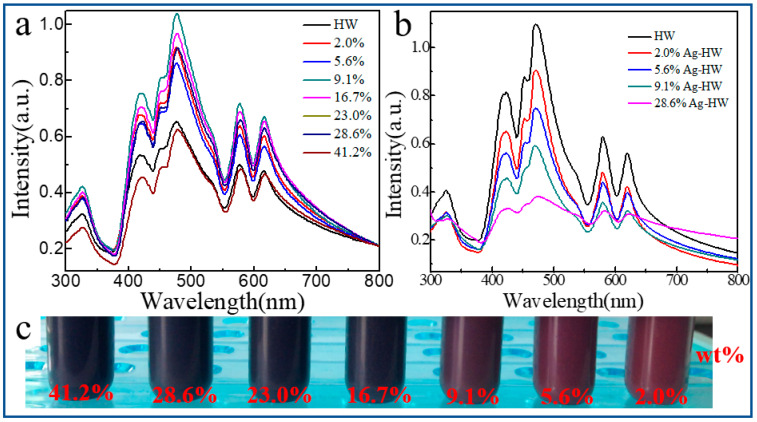
The absorption spectrum of M-HW nanocomposites with different metal NPs loadings ratios. (**a**) Pt-HW; (**b**) Ag-HW; (**c**) optical photographs of Pt-HW nanocomposites. All metal NPs loading proportions were represented in wt% unless otherwise stated.

The self-assembled porphyrin nanostructures exhibited a strong photoelectrochemical response in the visible light region (300–800 nm) owing to couple interactions and their ordered self-assembly arrays of porphyrin molecules through noncovalent interactions [51]. The UV–Visible absorption spectra of the resulting Pt-HW nanocomposites were shown in Figure 4a. The absorption of the porphyrin HWs had the Soret band at 420 nm and 477 nm, and the Q-band at 578 nm and 617 nm. The Pt-HW nanocomposites retained the absorption of the Soret band and the Q-band of HW. Moreover, the absorption intensity at 477 nm increased with the increase in the Pt loading amount. When the loading amount of Pt was 0.1 mg, the enhanced absorption reached the maximum value. Then, the absorption intensity gradually decreased, indicating that the excess Pt gradually covered the porphyrin HWs and blocked the surface absorption, suggesting that the Pt particles were evenly distributed on the surface of porphyrin HWs rather than agglomerated into large particles during the Pt reduction process. However, other precious metals, including Ag, Au, and Pd, did not have such features because the strong Ostwald ripening effect leaded to the reduced metal particles agglomerations into large particles on the surface of HW. The absorption intensity gradually decreased with the loading amount of Ag NPs (Figure 4b). The reaction solution of Pt-HW initially appeared to be purple under incandescent light due to light scattering by the porphyrin nanostructures. As the loading amount of Pt NPs increased, the dispersion of the Pt-HW nanomaterial gradually turned black (Figure 4c). Thus, the in situ photocatalytic synthesis method of SPR-based metal semiconductors M-HW is a promising photovoltaic technology aiming to emulate natural light harvesting processes and the energy storage.

### 3.3. Pt-HW Nanocomposites Photodegradation of Different Dyes

**Figure 5:**
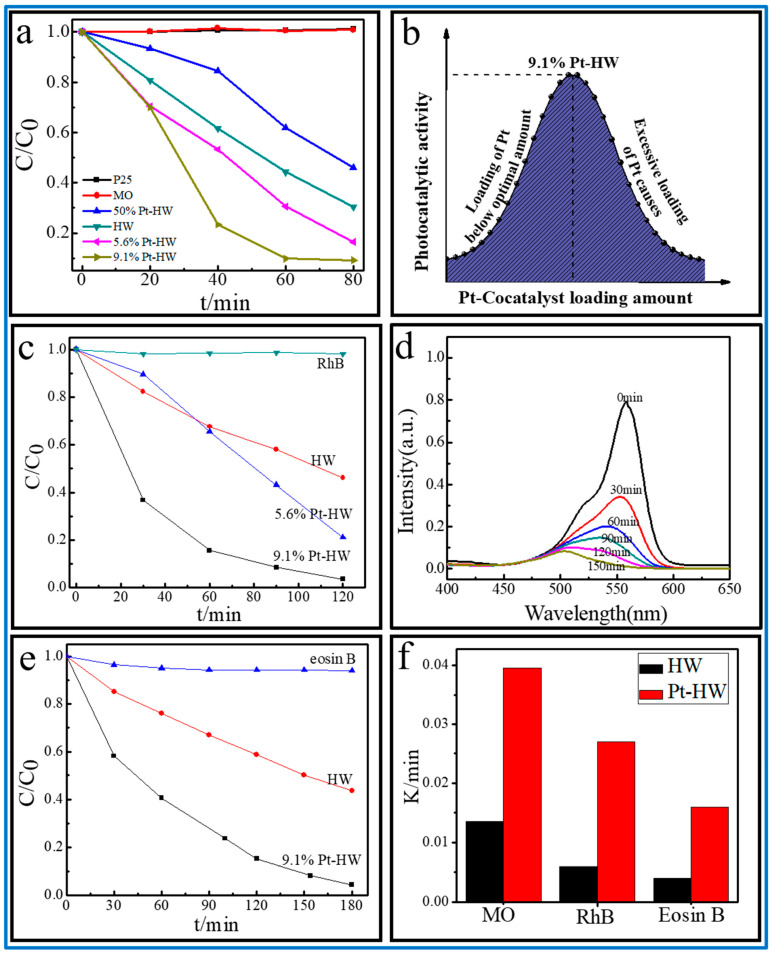
(**a**) MO degradation curves of Pt-HW with different Pt loadings; (**b**) degradation MO rate constant fitting map of Pt-HW with different Pt loading; (**c**) RhB degradation curve of Pt-HW; (**d**) UV–Vis absorption spectral changes for RhB as a function of irradiation time in the presence of 9.1% Pt-HW; (**e**) eosin B degradation curve of Pt-HW. (**f**) The comparison of photodegradation rate constant of different dyes using Pt-HW and HW.

Uniformly distributed SPR metal particle semiconductors (M-HW) enhanced the catalytic or chemical properties of the organic functional nanostructures, such as the visible light photocatalytic degradation of the organic pollutants. The Pt-HW nanocomposites were used to photodegrade different organic pollutants, including MO, rhodamine B (RhB), and eosin B. A series of degradation curves with different Pt loadings on different organic substrates were obtained (Figure 5). Several interesting catalytic features were observed: (1) when 0.1 mg of Pt was loaded on the surface of 1 mg of HW, the photocatalytic degradation performance of MO of 9.1% Pt-HW was three times that of HW (Figure 5a). (2) The photocatalytic performance gradually increased with increasing the Pt loading and became a plateau at 9.1 wt% Pt. However, excessive Pt loading hindered the absorption and the light irradiation, thus decreasing the catalytic effects of the photocatalysts (Figure 5b). (3) Then, the photocatalytic performances of Pt-HW were evaluated by degrading rhodamine B, and the photocatalytic degrading rate of RhB of 9.1% Pt-HW was improved by four times that of HW (Figure 5c). The absorbance of RhB almost declined to 0 after 2 h, indicating the high photocatalytic activity of the photocatalyst (Figure 5d). (4) The photocatalytic performance of degrading eosin B was improved by 2.6 times that of HW (Figure 5e). (5) Figure 5f compares the photodegradation activity of different dyes of the prepared samples. Pt-HW (9.1 wt%) exhibited an enhanced photocatalytic activity compared with HW and exhibited the substrates-dependent photocatalytic activity of different dyes. We attempted to correlate the relationship between Pt-HW and different substrates to study the cocatalyst pairs of metal particles in the porphyrin assemblies to provide additional surface sites, enhance the probability of photogenerated charge transport separation in the photocatalytic process, and improve the effect of the interface charge-transfer efficiency because of the SPR effect.

**Figure 6:**
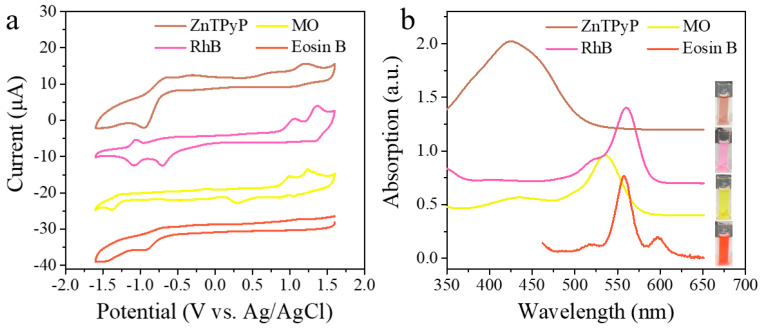
(**a**) Cyclic voltammograms of different dyes (test conditions: 0.1 M TBAPF_6_ in methylene chloride solution as an electrolyte solution). (**b**) The UV−Visible absorption spectrum of different dyes.

The experimental results showed that the higher the HOMO orbit energy, the easier it loses electrons [52,53,54]. Porphyrins are a class of organic complexes with unique macrocyclic conjugated structures. ZnTPyP molecules had higher HOMO orbital energy levels than the dye molecules (Figure 6 and Table 1). The ZnTPyP molecules in HW were more likely to lose electrons and create photogenerated holes under UV–Vis light radiation. The holes had strong oxidizing effects and captured the electrons of the dye molecules in the excited state. The excitation electrons of the HW combined with oxygen to produce superoxide radicals, which evolved into hydroxyl radicals, further leading to the degradation of the dyes [55]. The different oxidation potentials of the organic dyes and the complexity of the molecular structures led to different enhancement effects of the same catalyst on different dyes.

### 3.4. M-HW (M = Ag, Au, and Pd) Effects on the Enhancement of Photodegradation

**Figure 7:**
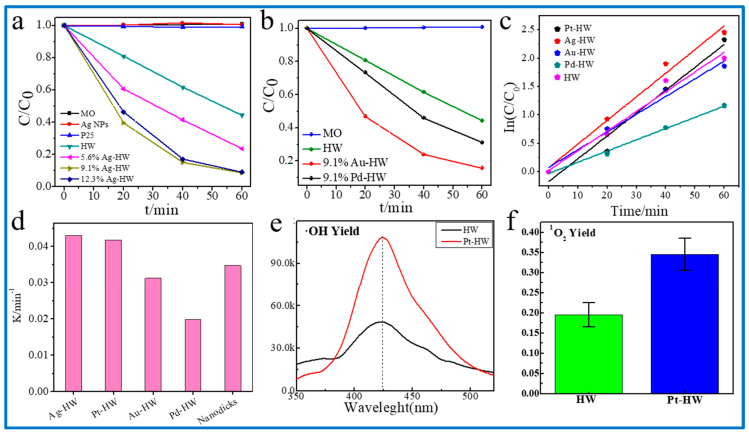
The degradation curves of MO with different metal loadings: (**a**) Ag-HW; (**b**) Au-HW; and Pd-HW. Comparison of rate constants of MO degradation using different composites at the same metal loading: (**c**) kinetics curves; (**d**) rate constants. The reactive oxygen species (ROS) yields of Pt-HW and HW under light irradiation: (**e**) hydroxyl radicals (·OH); (**f**) singlet oxygen (^1^O_2_).

The photocatalytic degradation curves of Ag-HW (Figure 7a), Au-HW, and Pd-HW (Figure 7b) nanocomposites were obtained. The first order kinetics of the visible light photocatalytic degradation curve of MO (Figure 7c) using different nanocomposites, namely Ag-HW, Au-HW, and Pd-HW, of the same metal loading were performed using Equation (1). Under the same SPR metal loading (0.1 mg), the photocatalytic performance of Ag-HW toward MO degradation was the best among the nanocomposites. The photocatalytic performance of k_Ag-HW_ (0.043 min^−1^) increased by three times that of HW, followed by k_Pt-HW_ (0.0417 min^−1^) > k_Au-HW_ (0.0312 min^−1^) > k_Pd-HW_(0.0198 min^−1^) > k_HW_ (0.014 min^−1^), suggesting that the M-HW degradation efficiency was higher than that of HW (Figure 7d). These results showed that M-HW exhibits a good enhancement effect. The result of k_Ag-HW_ and k_Pt-HW_ were 24% and 20% higher than that of k_nanodisc_ (0.0347 min^−1^), respectively.

The above experimental results showed that the Ag-HW nanocomposite had the best photocatalytic degradation performance among the other composites. On the one hand, the surface plasma enhancement effect of Ag enhanced the separation of the charge. On the other hand, a small amount of the Ag_2_S/Ag heterojunction was formed after the photoreduction process of silver-(I) thiosulfate used as a precursor [56]. We further detected the hydroxyl radicals (·OH) and singlet oxygen (^1^O_2_) yield of the prepared samples under light irradiation. After Pt NPs were loaded on HW, the productions of ·OH (Figure 7e) and ^1^O_2_ (Figure 7f) using Pt-HW were almost twice of using HW, which further confirmed the excellent catalytic activity of the SPR photocatalysts.

## 4. Photocatalytic Degradation Analysis

**Figure 8:**
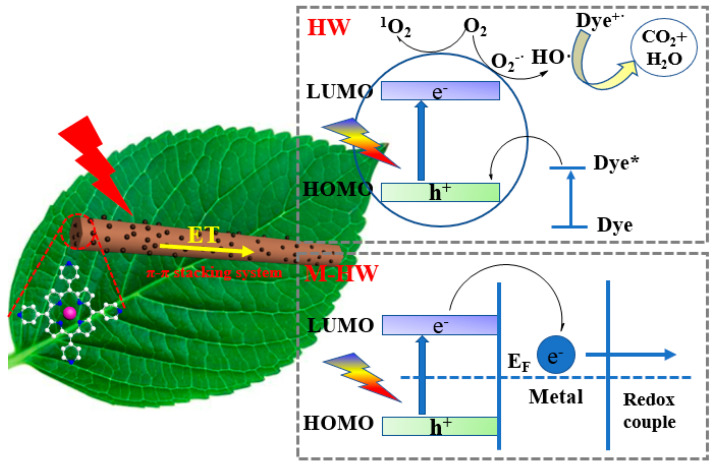
The charge-transfer process and mechanism of enhancing the photocatalytic performance.

The large π-electron conjugated systems and close packing between zinc porphyrin molecules endowed them with long-wave absorption, the low energy level of the molecules, and the charge transfer between the molecules. The HW photodegradation of the organic dyes underwent three steps (Figure 8): first, the zinc porphyrin molecule was irradiated under visible light, the electrons in the HOMO orbital of HW were excited and transferred to the LUMO orbital, and the electrons in the LUMO orbital combined with the oxygen in the air to generate superoxide radicals, which in turn evolved into hydroxyl radicals and further degraded the dye. However, the holes had oxidizing properties to despoil the electrons of the dye molecule, resulting in the continuous oxidization and decomposition of the dye molecules. The different oxidation potentials of the organic dyes and the complexity of the molecular structure enhanced the catalytic effect of the catalyst on different dyes. When certain amounts of precious metals (including Ag, Au, Pt, and Pd) were loaded on the surface of HW, the M-HW photodegradation of organic dyes underwent three steps (Figure 8). The improved efficiency of the visible light photocatalytic degradation of the organic dyes could be attributed to the following reasons: (1) these precious metal nanoparticles had SPR in the visible light region, which enhanced the light absorption of the porphyrin organic functional nanostructures, improved the energy conversion efficiency, and resulted in a good photostability, and (2) the electron interactions between precious metals nanoparticles and organic functional nanomaterials formed a higher Schottky barrier, which could effectively prevent the recombination of the photogenerated electrons and holes, and change the transmission route of the photogenerated electrons, thereby promoting the interface electrons. The charge transfer process enhanced the photocatalytic activity of the porphyrin self-assemblies [57,58,59,60]. (3) The work functions of Ag, Au, Pd, and Pt were 4.26, 5.1, 5.12, and 5.65 eV, respectively; that is, the Schottky barrier height after the HW was loaded with different metals (Ag < Au< Pd < Pt), which was responsible for the differences in the photocatalytic performance. However, the catalytic properties of the experiment did not correspond with these results due to the size of the metal particles, and the Pt and Ag nanoparticles were smaller in size, thus exhibiting better photocatalytic properties. This result showed that a substantial part of the electrons from the LUMO of HW was injected into the M NPs through the SPR excitation, which significantly enhanced the photogenerated charge transport separation and retarded the charge recombination during the photocatalytic process, thereby improving the visible light photocatalytic activity for the degradation of the organic pollutants. This result showed that the SPR metals provided an additional active site that enhanced the photogenerated charge transport separation during the photocatalytic process and improved the transfer efficiency of the interface charge. These findings provide an important reference into the application of organic-based SPR photocatalysis.

## 5. Conclusions

In summary, we successfully modified noble metal nanoparticles (Au, Ag, Pt, and Pd) on the surface of ZnTPyP HWs (M-HW) using an in situ photocatalytic method. The prepared M-HW nanocomposites photocatalysts were used to photodegrade different organic pollutants, and the results showed the photocatalytic degradation constant of k_Ag-HW_ (0.043 min^−1^) was the highest, followed by k_Pt-HW_ (0.0417 min^−1^) > k_Au-HW_ (0.0312 min^−1^) > k_Pd-HW_ (0.0198 min^−1^) > k_HW_ (0.014 min^−1^) under the same loading conditions (0.1 mg). The degradation efficiency of M-HW was superior to that of nanodisc, indicating that M-HW had the best photocatalytic degradation activity. The result of k_Ag-HW_ and k_Pt-HW_ was 24% and 20% higher than that of k_nanodisc_ (0.0347 min^−1^), respectively. These precious metal nanoparticles had an SPR in the visible light region, which enhanced the visible light absorption of porphyrin self-assemblies. The special base barrier enhanced the separation of the photogenerated charge during the photocatalytic process and improved the interface charge-transfer efficiency, thereby enhancing the photocatalytic activity of the porphyrin self-assemblies. As a novel technology, surface plasmon was first applied to porphyrin self-assemblies, which increased their light absorption and improved the energy conversion efficiency without changing the organic nanostructure. In addition, it has a unique advantage, making it a promising technology for preparing self-assembled nanostructures for photovoltaic and energy storage devices.

## Figures and Tables

**Table 1:** Electrochemical starting potential and electronic energy level table of zinc porphyrin and different dyes.

Compound	λ_max_ (nm)	E_g_^opt^ (eV)	E^ox^	E_HOMO_ (eV)	E_LUMO_ (eV)
ZnTPyP	624 nm	1.99	0.73 V	−5.45	−3.46
RhB	596 nm	2.08	1.05 V	−5.77	−3.69
MO	513 nm	2.42	1.01 V	−5.73	−3.31
Eosin B	578 nm	2.14	1.12 V	−5.84	−3.70

## Data Availability

Not applicable.

## References

[1] Marin M.L., Santos-Juanes L., Arques A., Amat A.M., Miranda M.A. (2012). Organic photocatalysts for the oxidation of pollutants and model compounds. Chem. Rev..

[2] Zhang N., Han C., Fu X., Xu Y.-J. (2018). Function-Oriented Engineering of Metal-Based Nanohybrids for Photoredox Catalysis: Exerting Plasmonic Effect and Beyond. Chem.

[3] Chen Y., Guerin S., Yuan H., O’Donnell J., Xue B., Cazade P.A., Haq E.U., Shimon L.J.W., Rencus-Lazar S., Tofail S.A.M. (2022). Guest Molecule-Mediated Energy Harvesting in a Conformationally Sensitive Peptide-Metal Organic Framework. J. Am. Chem. Soc..

[4] Dahl M., Liu Y., Yin Y. (2014). Composite titanium dioxide nanomaterials. Chem. Rev..

[5] Smith J.G., Faucheaux J.A., Jain P.K. (2015). Plasmon resonances for solar energy harvesting: A mechanistic outlook. Nano Today.

[6] Liu L., Zhang X., Yang L., Ren L., Wang D., Ye J. (2017). Metal nanoparticles induced photocatalysis. Natl. Sci. Rev..

[7] Ren H., Yang J.-L., Yang W.-M., Zhong H.-L., Lin J.-S., Radjenovic P.M., Sun L., Zhang H., Xu J., Tian Z.-Q. (2021). Core-Shell-Satellite Plasmonic Photocatalyst for Broad-Spectrum Photocatalytic Water Splitting. ACS Mater. Lett..

[8] Chen Y., Wang Y., Li W., Yang Q., Hou Q., Wei L., Liu L., Huang F., Ju M. (2017). Enhancement of photocatalytic performance with the use of noble-metal-decorated TiO_2_ nanocrystals as highly active catalysts for aerobic oxidation under visible-light irradiation. Appl. Catal. B Environ..

[9] Wang Y., Chen Y., Hou Q., Ju M., Li W. (2019). Coupling Plasmonic and Cocatalyst Nanoparticles on N(-)TiO_2_ for Visible-Light-Driven Catalytic Organic Synthesis. Nanomaterials.

[10] Wang C., Astruc D. (2014). Nanogold plasmonic photocatalysis for organic synthesis and clean energy conversion. Chem. Soc. Rev..

[11] Neyts E.C., Ostrikov K.K., Sunkara M.K., Bogaerts A. (2015). Plasma Catalysis: Synergistic Effects at the Nanoscale. Chem. Rev..

[12] Lee J.B., Choi S., Kim J., Nam Y.S. (2017). Plasmonically-assisted nanoarchitectures for solar water splitting: Obstacles and breakthroughs. Nano Today.

[13] Naya S.-I., Akita A., Morita Y., Fujishima M., Tada H. (2022). Crystallographic interface control of the plasmonic photocatalyst consisting of gold nanoparticles and titanium(iv) oxide. Chem. Sci..

[14] Ha H.D., Yan C., Katsoukis G., Kamat G.A., Moreno-Hernandez I.A., Frei H., Alivisatos A.P. (2020). Precise Colloidal Plasmonic Photocatalysts Constructed by Multistep Photodepositions. Nano Lett..

[15] Yuan L., Lou M., Clark B.D., Lou M., Zhou L., Tian S., Jacobson C.R., Nordlander P., Halas N.J. (2020). Morphology-Dependent Reactivity of a Plasmonic Photocatalyst. ACS Nano.

[16] Pu F., Huang Y., Yang Z., Qiu H., Ren J. (2018). Nucleotide-Based Assemblies for Green Synthesis of Silver Nanoparticles with Controlled Localized Surface Plasmon Resonances and Their Applications. ACS Appl. Mater. Interfaces.

[17] Ashok Kumar E., Wang T.-J., Chi H.-A., Chang Y.-H. (2022). Hydrothermal and photoreduction synthesis of nanostructured α-Fe_2_O_3_/Ag urchins for sensitive SERS detection of environmental samples. Appl. Surf. Sci..

[18] Liang X., Wang P., Gao Y., Huang H., Tong F., Zhang Q., Wang Z., Liu Y., Zheng Z., Dai Y. (2020). Design and synthesis of porous M-ZnO/CeO_2_ microspheres as efficient plasmonic photocatalysts for nonpolar gaseous molecules oxidation: Insight into the role of oxygen vacancy defects and M=Ag, Au nanoparticles. Appl. Catal. B Environ..

[19] Zhou X., Jin B., Luo J., Ning X., Zhan L., Xu X., Fan X., Yang F., Zhang S. (2019). One-pot solvothermal synthesis of 1D plasmonic TiO_2_@Ag nanorods with enhanced visible-light photocatalytic performance. Int. J. Hydrogen Energy.

[20] Cui Z., Wang W., Zhao C., Chen C., Han M., Wang G., Zhang Y., Zhang H., Zhao H. (2018). Spontaneous Redox Approach to the Self-Assembly Synthesis of Au/CeO_2_ Plasmonic Photocatalysts with Rich Oxygen Vacancies for Selective Photocatalytic Conversion of Alcohols. ACS Appl. Mater. Interfaces.

[21] Shang H., Huang S., Li H., Li M., Zhao S., Wang J., Ai Z., Zhang L. (2020). Dual-site activation enhanced photocatalytic removal of no with Au/CeO_2_. Chem. Eng. J..

[22] Veziroglu S., Obermann A.-L., Ullrich M., Hussain M., Kamp M., Kienle L., Leißner T., Rubahn H.-G., Polonskyi O., Strunskus T. (2020). Photodeposition of Au Nanoclusters for Enhanced Photocatalytic Dye Degradation over TiO_2_ Thin Film. ACS Appl. Mater. Interfaces.

[23] Yoshida T., Misu Y., Yamamoto M., Tanabe T., Kumagai J., Ogawa S., Yagi S. (2020). Effects of the amount of Au nanoparticles on the visible light response of TiO_2_ photocatalysts. Catal Today.

[24] Han N.S., Kim D., Lee J.W., Kim J., Shim H.S., Lee Y., Lee D., Song J.K. (2016). Unexpected Size Effect Observed in ZnO-Au Composite Photocatalysts. ACS Appl. Mater. Interfaces.

[25] Vamvasakis I., Liu B., Armatas G.S. (2016). Size Effects of Platinum Nanoparticles in the Photocatalytic Hydrogen Production Over 3D Mesoporous Networks of CdS and Pt Nanojunctions. Adv. Funct. Mater..

[26] Ma J., Tan X., Zhang Q., Wang Y., Zhang J., Wang L. (2021). Exploring the Size Effect of Pt Nanoparticles on the Photocatalytic Nonoxidative Coupling of Methane. ACS Catal..

[27] Hu R., Liao G., Huang Z., Qiao H., Liu H., Shu Y., Wang B., Qi X. (2021). Recent advances of monoelemental 2D materials for photocatalytic applications. J. Hazard. Mater..

[28] Li H., Wang L., Yu G. (2021). Covalent organic frameworks: Design, synthesis, and performance for photocatalytic applications. Nano Today.

[29] Zhong Y., Liu S., Wang J., Zhang W., Tian T., Sun J., Bai F. (2020). Self-assembled supramolecular nanostructure photosensitizers for photocatalytic hydrogen evolution. APL Mater..

[30] Wang L., Zhong Y., Sun J., Zhang F., Bai F. (2022). Controllable self-assembly of porphyrins and their applications. Sci. Sin. Chim..

[31] Cao R., Wang G., Ren X., Duan P.C., Wang L., Li Y., Chen X., Zhu R., Jia Y., Bai F. (2022). Self-Assembled Porphyrin Nanoleaves with Unique Crossed Transportation of Photogenerated Carriers to Enhance Photocatalytic Hydrogen Production. Nano Lett..

[32] Cao R., Wang J., Li Y., Sun J., Bai F. (2022). Morphology-controlled porphyrin nanocrystals with enhanced photocatalytic hydrogen production. Nano Res..

[33] Mandal S., Nayak S.K., Mallampalli S., Patra A. (2014). Surfactant-Assisted Porphyrin Based Hierarchical Nano/Micro Assemblies and Their Efficient Photocatalytic Behavior. ACS Appl. Mater. Interfaces.

[34] Zhong Y., Wang J., Zhang R., Wei W., Wang H., Lü X., Bai F., Wu H., Haddad R., Fan H. (2014). Morphology-Controlled Self-Assembly and Synthesis of Photocatalytic Nanocrystals. Nano Lett..

[35] Zhang N., Wang L., Wang H., Cao R., Wang J., Bai F., Fan H. (2018). Self-Assembled One-Dimensional Porphyrin Nanostructures with Enhanced Photocatalytic Hydrogen Generation. Nano Lett..

[36] Zhong Y., Wang J., Tian Y. (2019). Binary ionic porphyrin self-assembly: Structures, and electronic and light-harvesting properties. MRS Bull..

[37] Liu S., Li Z., Tong H., Zhong Y., Bai F., Hemali R., Gayani P. (2022). Porphyrin Self-Assembled Nanostructures and Applications. Self-Assembly of Materials and Supramolecular Structures.

[38] Geng G., Chen P., Guan B., Jiang L., Xu Z., Di D., Tu Z., Hao W., Yi Y., Chen C. (2017). Shape-Controlled Metal-Free Catalysts: Facet-Sensitive Catalytic Activity Induced by the Arrangement Pattern of Noncovalent Supramolecular Chains. ACS Nano.

[39] Bai F., Sun Z., Wu H., Haddad R.E., Xiao X., Fan H. (2011). Templated photocatalytic synthesis of well-defined platinum hollow nanostructures with enhanced catalytic performance for methanol oxidation. Nano Lett..

[40] Bai F., Sun Z., Wu H., Haddad R.E., Coker E.N., Huang J.Y., Rodriguez M.A., Fan H. (2011). Porous one-dimensional nanostructures through confined cooperative self-assembly. Nano Lett..

[41] Wang Z., Ho K.J., Medforth C.J., Shelnutt J.A. (2006). Porphyrin Nanofiber Bundles from Phase-Transfer Ionic Self-Assembly and Their Photocatalytic Self-Metallization. Adv. Mater..

[42] Zhong Y., Wang Z., Zhang R., Bai F., Wu H., Haddad R., Fan H. (2014). Interfacial Self-Assembly Driven Formation of Hierarchically Structured Nanocrystals with Photocatalytic Activity. ACS Nano.

[43] Wang Z., Medforth C.J., Shelnutt J.A. (2004). Self-Metallization of Photocatalytic Porphyrin Nanotubes. J. Am. Chem. Soc..

[44] Chankhanittha T., Yenjai C., Nanan S. (2022). Utilization of formononetin and pinocembrin from stem extract of Dalbergia parviflora as capping agents for preparation of ZnO photocatalysts for degradation of RR141 azo dye and ofloxacin antibiotic. Catal Today.

[45] Chen Y., Yan C., Dong J., Zhou W., Rosei F., Feng Y., Wang L.N. (2021). Structure/Property Control in Photocatalytic Organic Semiconductor Nanocrystals. Adv. Funct. Mater..

[46] Zhang Z., Zhu Y., Chen X., Zhang H., Wang J. (2019). A Full-Spectrum Metal-Free Porphyrin Supramolecular Photocatalyst for Dual Functions of Highly Efficient Hydrogen and Oxygen Evolution. Adv. Mater..

[47] Yang J., Jing J., Zhu Y. (2021). A Full-Spectrum Porphyrin-Fullerene D-A Supramolecular Photocatalyst with Giant Built-In Electric Field for Efficient Hydrogen Production. Adv. Mater..

[48] Jing J., Yang J., Li W., Wu Z., Zhu Y. (2022). Construction of Interfacial Electric Field via Dual-Porphyrin Heterostructure Boosting Photocatalytic Hydrogen Evolution. Adv. Mater..

[49] Wang J., Zhong Y., Wang L., Zhang N., Cao R., Bian K., Alarid L., Haddad R.E., Bai F., Fan H. (2016). Morphology-Controlled Synthesis and Metalation of Porphyrin Nanoparticles with Enhanced Photocatalytic Performance. Nano Lett..

[50] Nguyen Q.N., Wang C., Shang Y., Janssen A., Xia Y. (2022). Colloidal Synthesis of Metal Nanocrystals: From Asymmetrical Growth to Symmetry Breaking. Chem. Rev..

[51] Zhao Y., Hu Y., Zhong Y., Wang J., Liu Z., Bai F., Zhang D. (2021). Missing Links between the Structures and Optical Properties of Porphyrin Assemblies. J. Phys. Chem. C.

[52] Koposova E., Liu X., Pendin A., Thiele B., Shumilova G., Ermolenko Y., Offenhäusser A., Mourzina Y. (2016). Influence of Meso-Substitution of the Porphyrin Ring on Enhanced Hydrogen Evolution in a Photochemical System. J. Phys. Chem. C.

[53] He B., Zhang B.A., Liu F., Navarro A., Fernández-Liencres M.P., Lu R., Lo K., Chen T.L., Russell T.P., Liu Y. (2015). Electronic and Morphological Studies of Conjugated Polymers Incorporating a Disk-Shaped Polycyclic Aromatic Hydrocarbon Unit. ACS Appl. Mater. Interfaces.

[54] Natali M., Luisa A., Iengo E., Scandola F. (2014). Efficient photocatalytic hydrogen generation from water by a cationic cobalt(II) porphyrin. Chem. Commun..

[55] Guo P., Chen P., Ma W., Liu M. (2012). Morphology-dependent supramolecular photocatalytic performance of porphyrin nanoassemblies: From molecule to artificial supramolecular nanoantenna. J. Mater. Chem..

[56] Pollet B., Lorimer J.P., Phull S.S., Mason T.J., Walton D.J., Hihn J.Y., Ligier V., Wéry M. (1999). The effect of ultrasonic frequency and intensity upon electrode kinetic parameters for the Ag(S_2_O_3_)_23_−/Ag redox couple. J. Appl. Electrochem..

[57] Dasgupta N.P., Liu C., Andrews S., Prinz F.B., Yang P. (2013). Atomic layer deposition of platinum catalysts on nanowire surfaces for photoelectrochemical water reduction. J. Am. Chem. Soc..

[58] Lu Q., Lu Z., Lu Y., Lv L., Ning Y., Yu H., Hou Y., Yin Y. (2013). Photocatalytic Synthesis and Photovoltaic Application of Ag-TiO_2_ Nanorod Composites. Nano Lett..

[59] Bian Z., Tachikawa T., Zhang P., Fujitsuka M., Majima T. (2014). Au/TiO_2_ superstructure-based plasmonic photocatalysts exhibiting efficient charge separation and unprecedented activity. J. Am. Chem. Soc..

[60] Qian K., Sweeny B.C., Johnston-Peck A.C., Niu W., Graham J.O., DuChene J.S., Qiu J., Wang Y.C., Engelhard M.H., Su D. (2014). Surface plasmon-driven water reduction: Gold nanoparticle size matters. J. Am. Chem. Soc..

